# *OsELF3-1*, an Ortholog of *Arabidopsis EARLY FLOWERING 3*, Regulates Rice Circadian Rhythm and Photoperiodic Flowering

**DOI:** 10.1371/journal.pone.0043705

**Published:** 2012-08-17

**Authors:** Junming Zhao, Xi Huang, Xinhao Ouyang, Weilan Chen, Anping Du, Ling Zhu, Shiguang Wang, Xing Wang Deng, Shigui Li

**Affiliations:** 1 Rice Research Institute, Sichuan Agricultural University, Chengdu, Sichuan, China; 2 State Key Laboratory of Hybrid Rice, Sichuan Agricultural University, Chengdu, Sichuan, China; 3 College of Life Sciences, Beijing Normal University, Beijing, China; 4 Peking-Yale Joint Center for Plant Molecular Genetics and Agro-Biotechnology, National Laboratory of Protein Engineering and Plant Genetic Engineering, College of Life Sciences, Peking University, Beijing, China; Ohio State University, United States of America

## Abstract

*Arabidopsis thaliana EARLY FLOWERING 3* (*ELF3*) as a *zeitnehmer* (time taker) is responsible for generation of circadian rhythm and regulation of photoperiodic flowering. There are two orthologs (*OsELF3-1* and *OsELF3-2*) of *ELF3* in rice (*Oryza sativa*), but their roles have not yet been fully identified. Here, we performed a functional characterization of *OsELF3-1* and revealed it plays a more predominant role than *OsELF3-2* in rice heading. Our results suggest *OsELF3-1* can affect rice circadian systems via positive regulation of *OsLHY* expression and negative regulation of *OsPRR1, OsPRR37, OsPRR73* and *OsPRR95* expression. In addition, *OsELF3-1* is involved in blue light signaling by activating *EARLY HEADING DATE 1* (*Ehd1*) expression to promote rice flowering under short-day (SD) conditions. Moreover, *OsELF3-1* suppresses a flowering repressor *GRAIN NUMBER, PLANT HEIGHT AND HEADING DATE 7* (*Ghd7*) to indirectly accelerate flowering under long-day (LD) conditions. Taken together, our results indicate *OsELF3-1* is essential for circadian regulation and photoperiodic flowering in rice.

## Introduction

Flowering, referred to as heading date in rice (*Oryza sativa*), is an important agronomical trait for rice to adapt to specific cropping environments. Determined by photoreceptors, circadian clock systems and floral integrator genes, photoperiodic regulation is a key means in controlling plant flowering [1], [2], [3]. A sophisticated signaling network of photoperiodic flowering has been revealed initially based on the identification of key functional genes in *Arabidopsis thaliana* [4]. In the evolutionarily conserved GIGANTEA(GI)-CONSTANS(CO)-FLOWERING LOCUS T (FT) signaling pathways [3], clock-associated protein GI positively regulates *CO* expression under long-day conditions (LD) [5]. *CO* encodes a zinc-finger type transcriptional activator and promotes flowering under LD through up-regulation of its downstream target *FT*. FT as a floral activator controls flowering time by integrating input signals of various pathways [6], [7]. Interestingly, rice shares the similar pathway with *Arabidopsis thaliana* in photoperiodic flowering [8], [9]. Under short-day (SD) conditions, OsGI is an activator of *Heading date 1* (*Hd1*), the rice ortholog of *Arabidopsis CO* [10]; *Hd1* promotes flowering by activating *Heading date 3α* (*Hd3α*), the rice ortholog of *FT* [11], [12], [13]. Besides, *RICE FLOWERING LOCUS T 1* (*RFT1/FT-L3*) is extensively homologous to *Hd3α,* and these two homologs function redundantly in the flowering [14], [15].

Recently, many studies have indicated rice possesses a distinct flowering signaling machinery from *Arabidopsis*. There are some rice specific genes that have no orthologs in *Arabidopsis*, such as *EARLY HEADING DATE 1* (*Ehd1*), *Ehd2*, *Ehd3* and *GRAIN NUMBER, PLANT HEIGHT AND HEADING DATE 7* (*Ghd7*), play significant roles in photoperiodic flowering. *Ehd1* is a B-type response regulator and positively regulates *Hd3α* and *RFT* independently of *Hd1* [16]. *Ghd7* represses *Ehd1* expression to delay flowering under LD [17], [18]. *Ehd2* is an ortholog of maize (*Zea mays*) *INDETERMINATE 1* (*ID1*), and encodes a transcription factor for the upregulation of *Ehd1* [19]. *Ehd3* positively regulates *Ghd7* to repress heading under LD. On the contrary, *Ehd3* is independent of *Ghd7* and positively regulates *Ehd1* to promote flowering under SD [20]. Additionally, some MADS-box genes are crucial to rice photoperiodic flowering. *OsMADS50*, an ortholog of *Arabidopsis SUPPRESSOR OF OVEREXPRSSION OF CONSTANS1* (*SOC1*), acts in a dramatically distinct manner from *SOC1*. *SOC1* accumulates abundant mRNA and protein in the apical meristem and stimulates floral organ development downstream of *FT* [21], [22], [23]. However, *OsMADS50*, with a low expression level in the apical meristem, functions upstream of *Ehd1* to indirectly induce *RFT1* transcription under LD [24]. *OsMADS56,* another *SOC1* ortholog, attenuates *Ehd1* expression during LD [25]. *OsMADS51*, a rice-specific gene, also can positively regulate *Ehd1* under SD [25].

In *Arabidopsis*, *EARLY FLOWERING 3* (*ELF3*) is a *zeitnehmer* (time taker) to modulate resetting of the circadian clock and integrate temperature and photoperiod [26], [27], [28], [29], [30], [31]. Structurally, ELF3 exhibits no similarity to known functional factors. The C-terminal domain of ELF3 is required for its nuclear localization, while the N-terminus mediates the physical interaction of ELF3 with phytochrome B (phyB), a red light photoreceptor, and an E3 ubiquitin ligase CONSTITUTIVE PHOTOMORPHOGENIC1 (COP1) [32], [33], [34]. The ELF3-COP1 interaction enables ELF3 to associate with GI, and to further mediate GI degradation [34]. The central domain of ELF3 is necessary and sufficient to bridge EARLY FLOWERING 4 (ELF4) and a single-MYB domain-containing and SHAQYF-type GARP transcription factor LUX ARRHYTHMO (LUX) [35]. Mediated by LUX, the ELF4-ELF3-LUX complex directly binds to the promoters of *PHYTOCHROME INTERACTING FACTOR 4* (*PIF4*) and *PIF5* to regulate hypocotyl growth in the early evening, and the *PSEUDO-RESPONSE REGULATOR 9* (*PRR9*) promoter to sustain plant circadian rhythms [33], [36]. In rice, there are at least two orthologs of *Arabidopsis ELF3* [37], named as *OsELF3-1* (*LOC_Os06g05060*) and *OsELF3-2* (*LOC_Os01g38530*) hereafter. Late flowering was found in two mutants (NIL-*Hd17* and *ef7*) of *OsELF3-1* [38], [39] and a mutant (*osef3*) of *OsELF3-2* [40]. However, the roles of *OsELF3-1* and *OsELF3-2* have not yet been fully explored. In this study, we performed a functional characterization of *OsELF3-1* and found it plays a predominant role over *OsELF3-2* in rice heading. Our results suggest *OsELF3-1* affects rice circadian system via positive regulation of *OsLHY* and negative regulation on *OsPRR1*, *OsPRR37*, *OsPRR73* and *OsPRR95*. In addition, *OsELF3-1* is involved in blue light-induced activation of *Ehd1* expression to promote flowering under SD. Moreover, *OsELF3-1* suppresses a flowering repressor *Ghd7* to indirectly accelerate flowering under LD conditions. Taken together, our results reveal *OsELF3-1* is essential for circadian rhythm regulation and photoperiodic flowering in rice.

## Results

### The *oself3-1* mutant shows late heading

**Figure 1:**
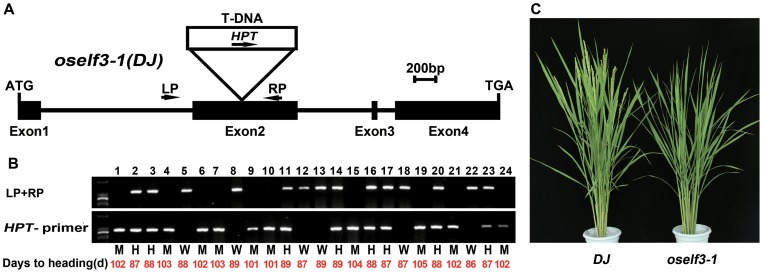
Schematic diagram of *OsELF3-1* gene structure, the T-DNA insertion site and *oself3-1* phenotype. (A) Schematic diagram of *OsELF3-1* gene structure and the T-DNA insertion site. Filled boxes indicate exons and solid lines indicate introns. Arrows indicate the primers used for analyzing the insertion site. LP and RP respectively represent the primers around the left and right borders of the T-DNA. (B) PCR genotyping of *OsELF3-1* segregants. W, wild type; M, homozygous; H, heterozygous. (C) Phenotypes of wild type and *oself3-1.* Photograph was taken when WT plant (*Dongjin*) flowered.

There are two putative orthologs of *Arabidopsis ELF3*, *OsELF3-1* and *OsELF3-2*, in the rice genome [37], [38], [39], [40]. OsELF3-1 and OsELF3-2 respectively share 35% and 30% identity with ELF3 in their amino acid sequences (Fig. S1). To investigate whether these two genes are involved in rice flowering, we examined the heading dates of *oself3-1* and *oself3-2* mutant in the experimental field of Sichuan province (30°67′) (summer) and Hainan province (18°15′) (winter) in China. A natural long-day condition (day length >14h) exists in Sichuan province in the summer, while a natural short-day condition (day length <10h) exists in Hainan province in winter. As shown in Fig. S2, two T-DNA insertion lines of *oself3-2* in *Zhonghua11* (*ZH11)* background and *Dongjin* (*DJ*) background both harbor a T-DNA fragment in the 4^th^ exon of *OsELF3-2*. These two lines did not show significantly different heading dates in either Sichuan or Hainan from wild-type plants (Table S1). However, *oself3-1* in *DJ* background with a T-DNA inserted into the 2^nd^ exon of *OsELF3-1* (Fig. 1A) delayed flowering 14 days in Sichuan (LD) (Fig. 1C) and 38 days in Hainan (SD) compared with *DJ* (Table 1). To confirm this phenotype of *oself3-1* is caused by the T-DNA insertion, we analyzed the co-segregation between the late heading defect and the existence of the T-DNA insertion. In 120 plants derived from the T2 T-DNA inserted population, two DNA markers were used to identify *HPT* and T-DNA insertion respectively. The correspondence between flowering phenotype and DNA markers suggested it is the T-DNA insertion that leads to a late flowering mutation (Fig. 1B).

**Table 1:** Days to heading in *oself3-1* and *OsELF3-1 RNAi* mutants.

Genotypes	Days to Heading under Wenjiang	Days to Heading under Hainan
WT(*DJ*)	87±0.9	65±1.5
*oself3-1*WT(*Ni*)*OsELF3-1 RNAi* Line1*OsELF3-1 RNAi* Line2*OsELF3-1 RNAi* Line3	101±1.594±2.0139±1.0143±1.6144±1.2	103±1.875±2.8137±1.3146±2.7149±1.6

Days to heading was calculated when the first panicle appeared. WT means wild type.

### *OsELF3-1 RNAi* plants flower late

**Figure 2:**
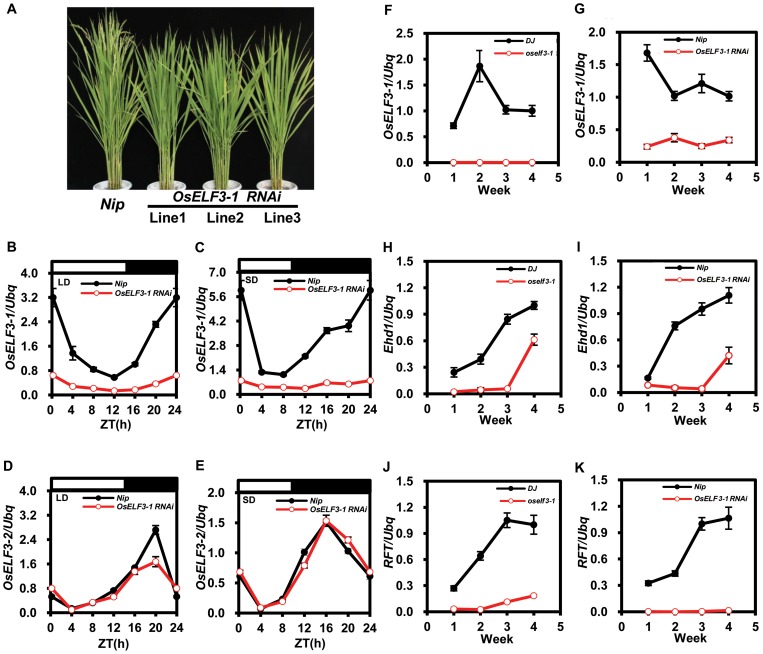
Phenotypes of *OsELF3-1 RNAi* lines and expression of flowering-related genes in *OsELF3-1 RNAi* mutant. (A) Flowering phenotypes of three independent *OsELF3-1 RNAi* lines. Photograph was taken when WT plant (*Nip*) flowered. (B–E) Diurnal expression of *OsELF3-1* and *OsELF3-2* in *Nip* and *OsELF3-1 RNAi* mutant under LD and SD. Rice leaves were harvested from 30-day old plants grown in the chambers at the indicated time points. White bars, light; black bars, darkness. ZT means Zeitgeber time. Values are means ± SD. (F–K) Expression of flowering-related genes in *OsELF3-1 RNAi* mutant. Rice leaves were harvested once a week at exactly the same time point for four weeks until the wild-type plants flowered. Values are means ± SD.

To further confirm the role of *OsELF3-1* in heading date, we constructed *OsELF3-1 RNAi* lines in *Nipponbare* (*Nip*) background. All the homozygous and stable T4 progenies of three independent transgenic lines consistently showed delayed flowering compared with *Nip* in both Sichuan and Hainan provinces (Table 1 and Fig. 2A). *OsELF3-1 RNAi* Line 1 was employed in our further experiments. Next, gene expression during a 24-hour period of *OsELF3-1* and *OsELF3-2* was examined in *OsELF3-1 RNAi* and *Nip* under SD and LD. We found in *OsELF3-1 RNAi*, *OsELF3-1* instead of *OsELF3-2* was constitutively repressed regardless of the conditions (Fig. 2B–E). Then we analyzed the *OsELF3-1* expression in *Nip* and *OsELF3-1 RNAi* using 60-day-old plants grown in an experimental field in Sichuan (LD) for another 4 weeks. Quantitative RT-PCR (qRT-PCR) results showed the *OsELF3-1* mRNA level was continuously suppressed in *OsELF3-1 RNAi* (Fig. 2F, G). Therefore, the late flowering phenotype in *OsELF3-1 RNAi* resulted from *OsELF3-1* gene repression.

In *Arabidopsis*, *elf3* mutants flower much earlier than wild-type plants under LD and SD conditions [26]. ELF3 indirectly represses *FT* expression through positively regulating degradation of GI under SD [34]. In rice, it was noted that RFT integrates all flowering signals and induces the development of floral organs under LD [3], so we next asked whether *RFT* and its regulator *Ehd1* are affected by *OsELF3-1*. Rice leaves were harvested once a week at exactly the same time point for four weeks until the wild-type plants flowered. We observed that the transcription of *Ehd1* maintained at a lower level in *oself3-1* and *OsELF3-1 RNAi* mutants than that in their wild-type counterparts (Fig. 2H, I). There was a notable increase in *RFT* expression in wild-type plants, whereas *RFT* expression was severely attenuated in *oself3-1* and *OsELF3-1 RNAi* mutants (Fig. 2J, K). Taken together, our results suggest that loss of *OsELF3-1* function leads to delayed flowering via repressing *Ehd1* and *RFT* under LD.

### *OsELF3-1* affects the expression of circadian clock components

Earlier studies demonstrated that florigen is the hypothetical floral signal that is produced in leaves [41]. Gene products of florigen, mature FT and Hd3α proteins, specifically translocate to shoot apical meristem (SAM) to induce flowering [13], [42]. We are also interested whether *OsELF3-1* exhibits organ-specific expression in rice. Using rice grown under natural LD at the booting stage, qRT-PCR analyses were performed with total RNA isolated from roots, stems, leaves, leaf sheaths and young panicles (Fig. S3). The transcript levels of *OsELF3-1* were highest in leaves and leaf sheaths tissues while lowest in roots and stems. *OsELF3-1* shares the similar tissue-specific expression profile with *Hd3α* and *RFT* in leaves and leaf sheaths, indicating *OsELF3-1* may play a vital role in flowering regulation. Previous reports have shown that *Arabidopsis ELF3* expression varies in the time span of one day, with a trough in the early day and a peak in the early night. Therefore *ELF3* was described as an evening-phased gene [43]. Intriguingly, *OsELF3-1* mRNA showed a peak at dawn (ZT0) under LD and SD (Fig. 2B, C). Accordingly, *OsELF3-1* is a morning-phased gene, suggesting a distinct role of *OsELF3-1* from *Arabidopsis ELF3*.

The core circadian clock components, which are connected by positive–negative feedback loops, form a sophisticated network in *Arabidopsis* [4], [44]. The central loop identified first contains two morning-phased transcription factors CIRCADIAN CLOCK ASSOCIATED 1 (CCA1) and LATE ELONGATED HYPOCOTYL (LHY), and an evening-phased gene PSEUDO-RESPONSE REGULATOR 1 (PRR1 or TOC1) [4]. In the morning, CCA1 and LHY, the transcriptional repressors, can associate with the promoter of *TOC1* and negatively regulate *TOC1* transcription [45], [46], [47], [48], [49]. TOC1 directly represses the expression of *CCA1* and *LHY*, through binding to the promoters of *CCA1* and *LHY* at night [50], [51]. ELF3 physically interacts with a transcription factor LUX to associate with the *PRR9* promoter and represses the transcription of *PRR9* [36], [52], [53]. Because PRR9 is a repressor of *CCA1* and *LHY* expression, ELF3 enables itself to indirectly activate *CCA1* and *LHY* [52]. It has been well documented that *OsLHY* and *OsPRR1* have similar circadian expression profiles with their *Arabidopsis* orthologs *CCA1*/*LHY* and *TOC1* respectively [37]. We next studied the effects of OsELF3-1 on the expression of clock-associated genes.

**Figure 3:**
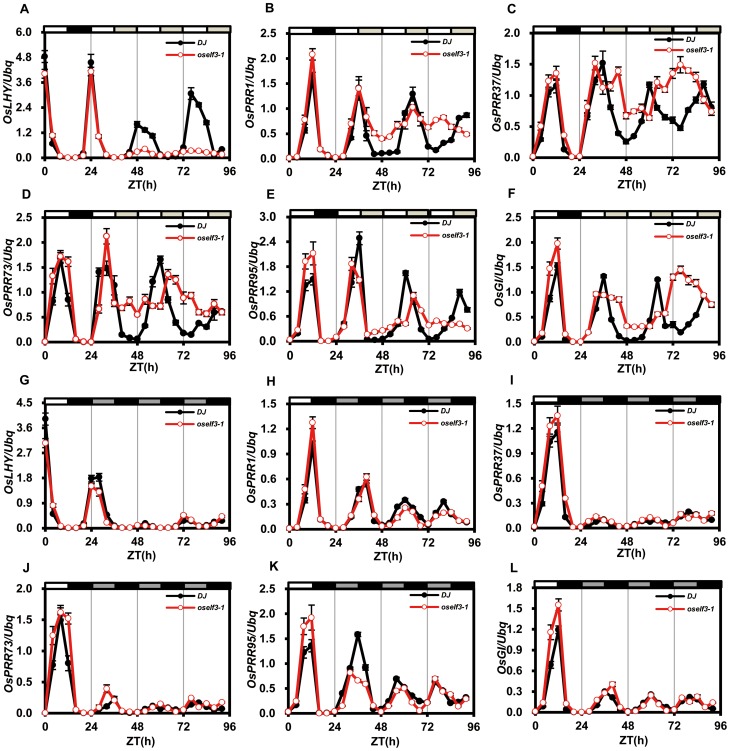
Circadian expression of clock-associated genes in *oself3-1*. Circadian expression of *OsLHY*, *OsPRR1*, *OsPRR37*, *OsPRR73*, *OsPRR95* and *OsGI* in *DJ* (filled circles) and *oself3-1* (open circles) under LL and DD. The plants were first grown to cycles of 12-hour light/12-hour darkness for 30 days at 26°C and then transferred to the continuous light (LL) or continuous darkness (DD) at dawn. In A–F panels, white bars, light; black, darkness; gray, subjective light. In G–L panels, white bars, light; black, darkness; gray, subjective darkness. ZT means Zeitgeber time. Values are means ± SD.

In *oself3-1* mutant, we found that the maintenance of the daily expression oscillation of *OsLHY* and *OsPRR1* was disrupted under continuous light (LL) rather than continuous darkness (DD) (Fig. 3A, B, G and H). Previously, Murakami *et al.* identified the rice orthologs of *Arabidopsis PRR3/PRR7* and *PRR5/PRR9*. Since it is difficult to distinguish the exact counterparts in rice, they were designated as *OsPRR37* and *OsPRR73*, and *OsPRR59* and *OsPRR95* [37], [54]. We then asked whether *OsPRR37*, *OsPRR73*, *OsPRR59* and OsPRR*95* transcription are regulated by *OsELF3-1*. The altered circadian profiles were observed in the expression of *OsPRR37*, *OsPRR73*, *OsPRR95* and *OsGI* (Fig. 3C–F and I–L) under LL rather than DD. The similar defects in the circadian rhythm were also shown in *OsELF3-1 RNAi* (Fig. S4).

**Figure 4:**
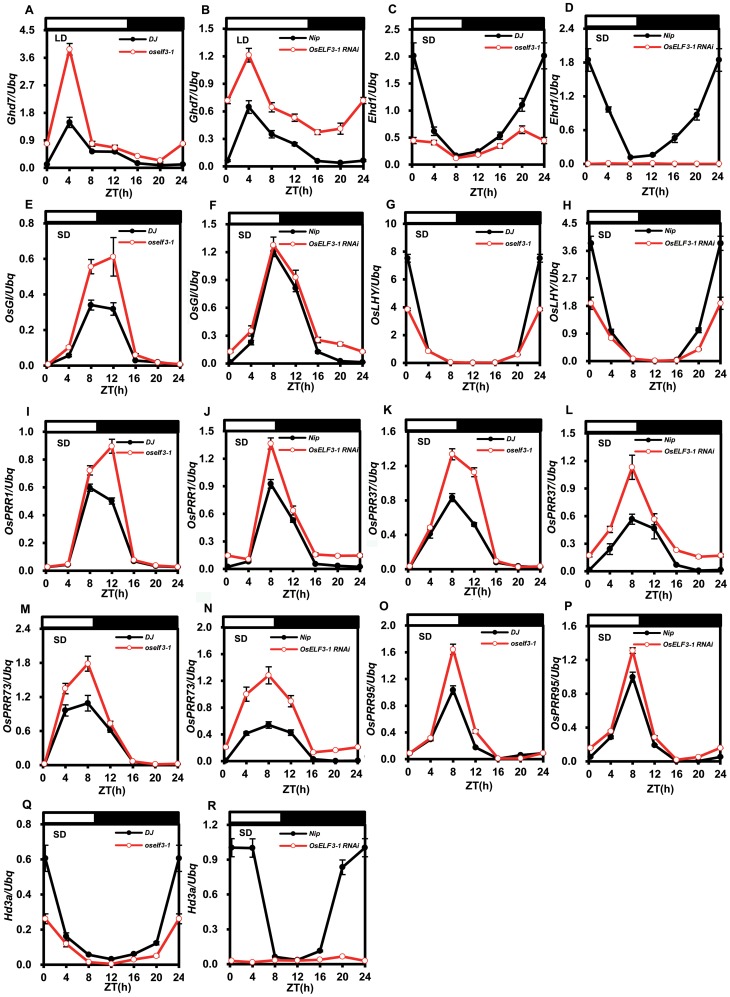
Diurnal expression of clock-associated genes in *oself3-1* and *OsELF3-1 RNAi* mutants. Diurnal expression patterns of *Ghd7*, *Ehd1*, *OsGI*, *OsLHY*, *OsPRR1*, *OsPRR37*, *OsPRR73*, *OsPRR95* and *Hd3α* in *DJ* (filled circles) and *oself3-1* (open circles), and in *Nip* (filled circles) and *OsELF3-1 RNAi* (open circles) plants. Rice leaves were harvested from 30-day old plants grown in the chambers at the indicated time points. White bars, light; black bars, darkness. ZT means Zeitgeber time. Values are means ± SD.

In addition, we found that the mutation of *OsELF3* led to the abnormal diurnal expression of these genes. *OsLHY* and *OsPRR1* transcription peaks appeared at dawn and dusk, respectively, in wild-type plants under SD (Fig. 4G–J). However, the expression altitude of *OsLHY* showed a significant reduction in *oself3-1* and *OsELF3-1 RNAi* under SD (Fig. 4G, H). *OsPRR1* transcrption level was higher at dusk in these mutants (Fig. 4I, J). Therefore, we suggested *OsELF3-1* can positively regulate *OsLHY* expression and negatively regulate *OsPRR1* expression. Meanwhile, *OsPRR37*, *OsPRR73* and *OsPRR95* showed higher expression peaks at dusk under SD in *oself3-1* and *OsELF3-1 RNAi* mutants than that in wild-type plants (Fig. 4K–P), suggesting *OsELF3-*1 negatively regulates *OsPRR37*, *OsPRR73* and *OsPRR95*.

### *OsELF3-1* activates flowering through a rice photoperiodic flowering pathway

It has been well documented that the mutants of core circadian clock genes in *Arabidopsis,* such as *cca1* and *lhy,* suffer from severe flowering defects [46], [47]. In contrast, there is no evidence showing mutants of core circadian clock genes in rice exhibit abnormal flowering phenotypes. It is still unknown how *oself3-1* and *OsELF3-1 RNAi* mutants delay flowering. Therefore, we analyzed whether *OsELF3-1* affects *Ghd7*, *Ehd1* and *Hd1* at the transcriptional level.

Xue *et al.* suggested *Ghd7* is a flowering repressor to negatively regulate *Ehd1* under LD [17]. *Ehd1*, an important flowering activator, promotes *RFT1* and *Hd3α* transcription under LD and SD respectively. The mRNA level of *Ehd1* peaks at dawn [3], [16]. We observed that *Ghd7* in *oself3-1* and *OsELF3-1 RNAi* mutants accumulates more transcripts than that in wild-type plants in the subject day under LD (Fig. 4A, B), as evidence that *OsELF3-1* represses *Ghd7* under LD. Moreover, under SD, *Ehd1* in *oself3-1* and *OsELF3-1 RNAi* mutants accumulates less transcripts than that in wild-type plants at dawn (Fig. 4C, D); *Hd3α* expression in *oself3-1* and *OsELF3-1 RNAi* mutants remained at a very low level over the 24-hour period (Fig. 4Q, R); *OsGI* expression level was elevated in *oself3-1* (Fig. 4E). Taken together, we proposed that *OsELF3-1* represses *Ghd7* expression in the morning under LD and activates *Ehd1* expression at dawn under SD.

### *OsELF3-1* is involved in the blue light-mediated activation of *Ehd1* expression

**Figure 5:**
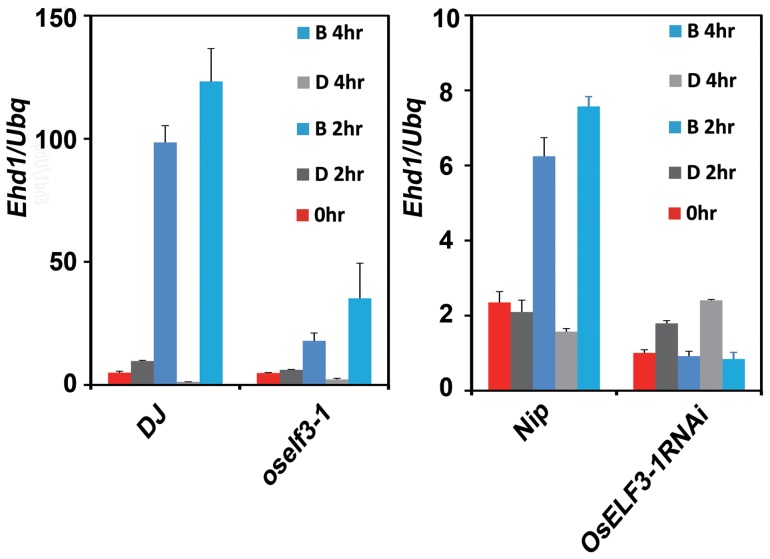
Blue light promotes activation of *Ehd1* dependent on *OsELF3-1*. Rice seedlings were first grown under SD for 14 days and then transferred to the blue light (75 μmol m^−2^ s^−1^) or darkness at dawn, and harvested at the indicated time points. Values are means ± SD.

In *Arabidopsis*, ELF3 is an adaptor to target GI to the E3 ubiquitin ligase COP1-mediated degradation. This process may be repressed by the physical interaction of blue light receptors cryptochromes (CRY) with COP1 under blue light [34]. It was also pointed out that *OsGI* is a gate of circadian clocks in response to blue light in the morning to activate *Ehd1* regardless of photoperiod in rice [18]. Since *OsELF3* is important for *Ehd1* expression (Fig. 4C, D), *OsELF3-1* induces *Ehd1* expression at dawn under SD, we examined whether *OsELF3-1* is involved in *Ehd1* activation in response to blue light in the morning. Wild type, *oself3-1* and *OsELF3-1 RNAi* plants were entrained for 14 days under SD conditions and then transferred to blue light and dark surroundings in the following morning. In wild-type plants, activation of *Ehd1* transcription was detectable within 2 hours after blue light treatment (Fig. 5). However, *Ehd1* gained little induction in *oself3-1* and *OsELF3-1 RNAi* mutants (Fig. 5). Thus, our results indicated *OsELF3-1* is essential for *Ehd1* activation in response to blue light in the morning under SD.

## Discussion

### *OsELF3-1* plays a pivotal role in rice flowering

Previous results show *OsELF3-1* and *OsELF3-2* (*OsEF3*) are involved in rice flowering [38], [39], [40]. However, their functions have not been fully explored. In our study, we obtained two T-DNA insertion lines of *OsELF3-2* in *ZH11* and *DJ* backgrounds. Each line has an insertion site in the 4^th^ exon which might cause an abnormal C-terminus of OsELF3-2 protein (Fig. S2). *In Arabidopsis*, the C-terminal domain of ELF3 is responsible for its nuclear localization [32], [33]. Since OsELF3 shares intensive amino acid similarity with *Arabidopsis* ELF3 especially in its C-terminal region, we speculated that OsELF3-2 might be misallocated in these mutants. Surprisingly, these mutants do not exhibit flowering defects (Table S1), whereas a T-DNA insertion line *oself3-1* showed a severe late flowering phenotype under both LD and SD (Fig. 1C, Table 1). Thus, we conclude that *OsELF3-1* probably plays a predominant role over *OsELF3-2* in rice flowering. Furthermore, *OsELF3-1* exhibits difference diurnal expression pattern with *Arabidopsis ELF3* [43] and *OsELF3-2* (Fig. 2B–E). It raises the possibility that OsELF3-1 might play a divergent role from *Arabidopsis* ELF3 and OsELF3-2 through sub-functionalization in the control of rice development.

In *Arabidopsis*, ELF3 activity is required to restrain expression of temperature-sensitive genes like *PRR7* and *PRR9* in the day phase, and to establish enhanced sensitivity of these genes to warm temperature cues during the night. It is necessary for the core oscillator to progress from day to night accompanied by temperature changes [28], [29]. ELF3 is also engaged in phyB-mediated responses to ambient temperature [30]. Recently, Kolmos *et al*. identified a dual role of ELF3 as an integrator of phytochrome signals and a repressor of core oscillator [55]. Thus it is interesting to explore the potential functions of OsELF3-1 in coordinating various environmental stimuli and circadian regulation in our future analysis.

### *OsELF3-1* is important for rice circadian clock

Earlier researchers have identified ELF3 is a *zeitnehmer* in affecting the signaling transduction from light input to oscillators and modulates circadian clock in *Arabidopsis* [28], [31]. Recently, Matsubara *et al*. found no obvious difference in the expression of clock-associated genes between *Nip* and NIL-*Hd17* [39]. Saito *et al*. reported Cab1R: LUC expression under DD conditions was not affected in the *oself3-1* (*ef7*) mutant, while the period of free-running rhythms was slightly shortened in the *oself3-1* under LL conditions [38]. However, our data clearly demonstrate *OsELF3-1* is important for the circadian expression of *OsLHY*, *OsPRR1*, *OsPRR37, OsPRR73 and OsPRR59* under free-running LL conditions, taking advantage of the *oself3-1* and *OsELF3-1 RNAi* mutants (Fig. 3A–E, Fig. S4). These results showed *OsELF3-1* is essential for sustaining the robust oscillations of *OsLHY* and *OsPRRs* expression. Furthermore, *OsLHY*, *OsPRR1*, *OsPRR37, OsPRR73 and OsPRR59* were not affected under DD in these two mutants, indicating that *OsELF3-1* might be responsible for mediating light input to the core circadian oscillator.

Recently, identification of a novel evening complex has profoundly elucidated the biochemical role of ELF3 in the maintenance of the circadian clock in *Arabidopsis* [33], [36]. ELF3 recruits ELF4 and LUX proteins to form a transcriptional-repressor complex that associates with the promoter of *PRR9*, a repressor of *CCA1* and *LHY* expression, and then down-regulates *PRR9* expression [33]. Thus, this complex indirectly activates *LHY* and *CCA1* in a double-negative connection via *PRR9* [33], [52]. Our results showed *OsELF3-1* positively regulates *OsLHY* at dawn (Fig. 4G, H), while it negatively regulates *OsPRR1, OsPRR37, OsPRR73* and *OsPRR59* at dusk (Fig. 4I–P). Therefore, we deduce OsELF3-1 might associate with the promoters of *OsPRR*s to repress *OsPRR*s in the early evening and to indirectly induce *OsLHY* at dawn.

### Functioning mode of *OsELF3-1* in rice photoperiodic flowering

Lately, Ioth *et al*. reported that a critical day length of less than 13 hours is essential for the initiation of rice photoperiodic flowering. The reduction of day length from 13.5 to 13 hours can activate *Ehd1* expression along with the observable increase in *Hd3α* mRNA accumulation. This process is triggered by blue light which coincides with the morning phase set by *OsGI*-dependent circadian clocks [18]. Our results showed blue light-induced expression of *Ehd1* is also dependent on *OsELF3-1* in the morning under SD (Fig. 4A, B). The observation of increased *OsGI* mRNA levels in *oself3-1* mutants under LL and SD conditions (Fig. 3F, 4E and F) gives rise to the possibility that *OsGI* might feedback on the reduced *Ehd1* expression to function in a compensatory way for the loss of *OsELF3-1.* It is also known that *Arabidopsis* ELF3 protein can physically interact with COP1 to mediate GI degradation *in vivo* [34]. Thus, further efforts will be made to elucidate the functional interaction between *OsELF3-1* and *OsGI* in rice flowering, particularly in *Ehd1* activation induced by blue light under SD.

It is worth pointing out that under LD, phytochromes play pivotal roles in controlling rice flowering. This fact can be explained mainly through two possible signaling pathways. Firstly, the light-dependent interaction between phyB and Hd1 attenuates the Hd1 activation activity on *Hd3α*, leading to low mRNA abundance of *Hd3α* in the evening under LD [56], [57]. Secondly, *Ghd7* repression on *Ehd1* in the morning also rests on phyB under LD [17], [58]. In our study, *OsELF3-1* promotes rice flowering under LD, via repressing *Ghd7* and indirectly activating *Ehd1* expression (Fig. 1C, 2A, H, I, 4A–D, Table 1 and Table S1). It is promising that *OsELF3-1* might be involved in phyB-mediated flowering regulation. Besides, in *Arabidopsis*, ELF3-phyB complex regulates early photomorphogenic development while ELF3 and phytochromes work in concert to control flowering via independent signaling pathways [32], [59], [60]. It is also of importance to explore the roles of OsELF3-1 in response to red light.

**Figure 6:**
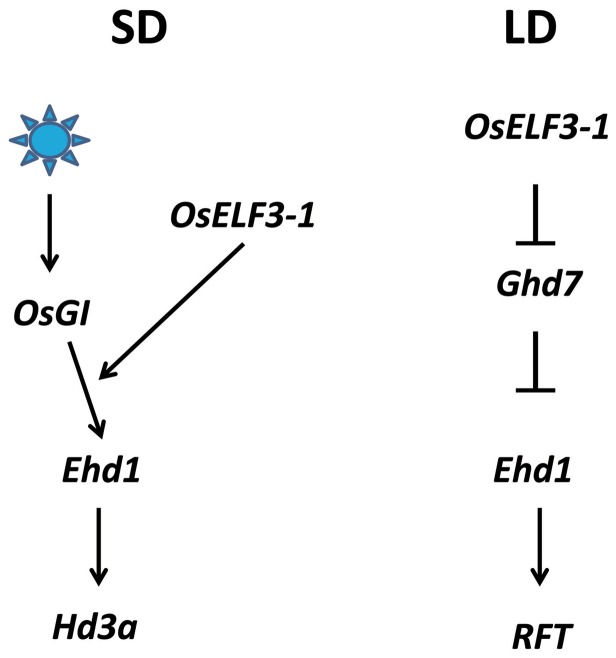
A working model for *OsELF3-1* in rice flowering. *OsELF3-1* is a flowering inducer via transcriptional regulation on *Ehd1* and *Ghd7*. The blue light-mediated activation of *Ehd1* expression is dependent on *OsELF3-1.* Arrows, positive regulation. Bars, negative regulation.

In summary, we point out *OsELF3-1* functionally associates with the rice circadian clock and control of photoperiodic flowering. *OsELF3-1* is essential for the expression of core oscillator genes and the maintenance of circadian clocks (Fig. 3 and Fig. S4). In photoperiodic flowering pathway, *OsELF3-1* is a flowering inducer via transcriptional regulation of *Ehd1* and *Ghd7* (Fig. 6).

## Materials and Methods

### Plant Materials and Growth Conditions

The T-DNA insertion mutants *oself3-1* and *oself3-2* (*DJ*) were obtained from the Salk Institute Genomic Analysis Laboratory http://signal.salk.edu/cgi-bin/RiceGE [61]. *oself3-*2 (*ZH11*) was isolated by screening a T-DNA insertion rice (*Oryza sativa*) var. *japonica* “*Zhonghua11*” population generated by Shigui Li's laboratory at Sichuan Agricultural university [40]. To make the *OsELF3-1 RNAi* construct, an *OsELF3-1* cDNA fragment (406–967 bp) was amplified and cloned into the *Xba*I/*Bam*HI and *Hind*III/*Sal*I sites of pBluescript SK- vector in sense/antisense directions respectively. The DNA fragment containing the sense/antisense *OsELF3-1* fragment was excised by *Bam*HI/*Sal*I and cloned in pOsActin2-1-nos binary vector [62], under the rice *Actin2* promoter. Rice plants were transformed according to *Agrobacterium* mediated co-cultivation methods [63]. All the primers used in this study were listed in Table S2.

Screening of the flowering mutants was carried out by growing the rice plants in the experimental field of Sichuan Agricultural University in Sichuan province, China, in summer (natural LD) and in Hainan province in winter (natural SD). For diurnal expression experiments, the plants were grown in chambers at 70% humidity under LD with daily cycles of 14-hour light at 26°C, 10-hour darkness at 22°C, or under SD with 10-hour light at 26°C and 14-hour darkness at 22°C. Light was provided by fluorescent white light tubes (400 to 700 nm, 100 μmol m^−2^ s^−1^). For circadian rhythm experiments, the plants were first grown to cycles of 12-hour light/12-hour darkness for 30 days at 26°C and then transferred to the continuous light (LL) or continuous darkness (DD) at dawn. For Blue light activation experiments, rice seedlings were first grown under SD for 14 days and then transferred to the blue light (75 μmol m^−2^ s^−1^) or darkness at dawn.

### RNA extraction and qRT-PCR

Total RNA was extracted using a RNeasy plant mini kit (Qiagen, USA), and treated with DNaseI (Qiagen, USA). cDNA was synthesized from 2 μg of total RNA using SuperScript III Reverse Transcriptase (Invitrogen, USA). The quantitative analysis of gene expression was performed with SsoFast EvaGreen PCR Master Mix (BIO-RAD, USA). Data were collected using the BIO-RAD CFX96 Real-Time PCR System according to the manufacturer's instructions. Each experiment was repeated with three independent samples, and Real-Time PCR was performed in three technical replicates for each sample. Changes in gene expression were calculated via the ΔΔ_Ct_ method [64]. The primers were listed in Table S2.

## Supporting Information

Figure S1 **Alignment of ELF3 orthologs from the model organisms.** CLC Protein Workbench 5 software was used in this analysis.
(TIF)

Figure S2 **Schematic diagram of** ***OsELF3-2*** **gene structure and the T-DNA insertion sites of** ***oself3-2*** **mutants.** Filled boxes indicate exons and solid lines indicate introns.
(TIF)

Figure S3 **Tissue-specific expression of** ***OsELF3-1.*** QRT-PCR analysis of *OsELF3-1* expression in different tissues. Rice were grown in Sichuan natural LD field and harvested at 9∶00 am at the booting stage. Values are means ± SD.
(TIF)

Figure S4 **Circadian expression of clock-associated genes in** ***Nip*** **and** ***OsELF3-1 RNAi.*** Circadian expression of *OsLHY*, *OsPRR1*, *OsPRR37*, *OsPRR73*, *OsPRR95* and *OsGI* in *Nip* (filled circles) and *OsELF3-1 RNAi* (open circles) under LL and DD. The plants were first grown to cycles of 12-hour light/12-hour darkness for 30 days at 26°C and then transferred to the continuous light (LL) or continuous darkness (DD) at dawn. In A-F panels, white bars, light; black, darkness; gray, subjective light. In G-L panels, white bars, light; black, darkness; gray, subjective darkness. ZT means Zeitgeber time. Values are means ± SD.
(TIF)

Table S1 **Days to heading in** ***oself3-2*** **plants in 2010 and 2011.** Days to heading was calculated when the first panicle appeared. WT means wild type.
(DOCX)

Table S2 **Primers used in this study.**
(DOCX)
