# Supplementary figures and images for: OsELF3-1, an Ortholog of Arabidopsis EARLY FLOWERING 3, Regulates Rice Circadian Rhythm and Photoperiodic Flowering
Source: PLoS One. 2012 Aug 17;7(8):e43705. doi: 10.1371/journal.pone.0043705 (PMC3422346; doi:10.1371/journal.pone.0043705)

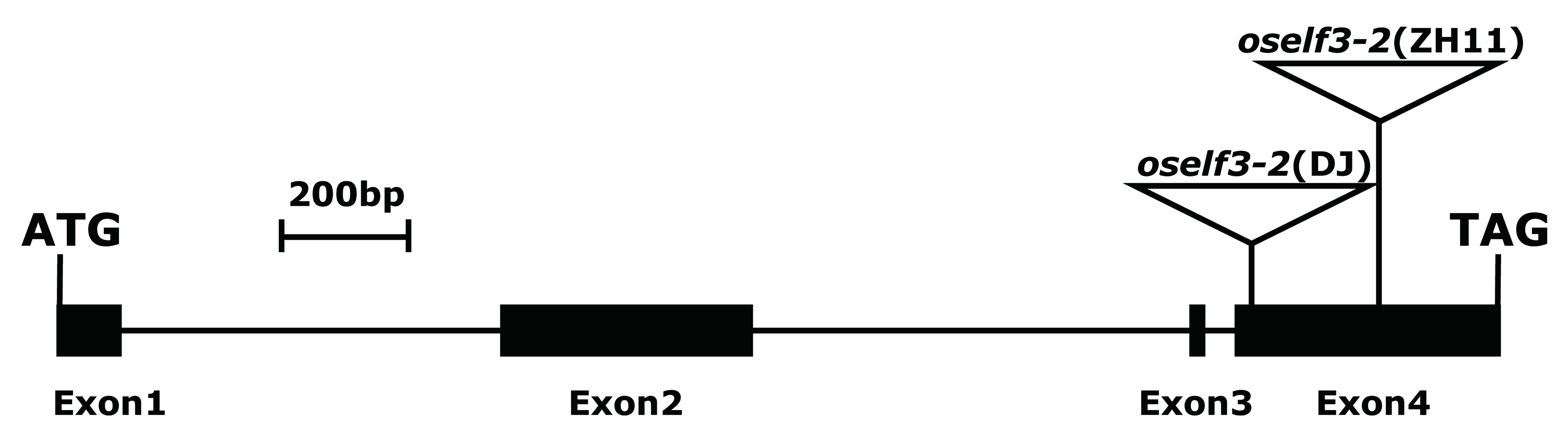

Supplement: Figure S2 — Schematic diagram of OsELF3-2 gene structure and the T-DNA insertion sites of oself3-2 mutants. Filled boxes indicate exons and solid lines indicate introns. (TIF) [file pone.0043705.s002.tif]

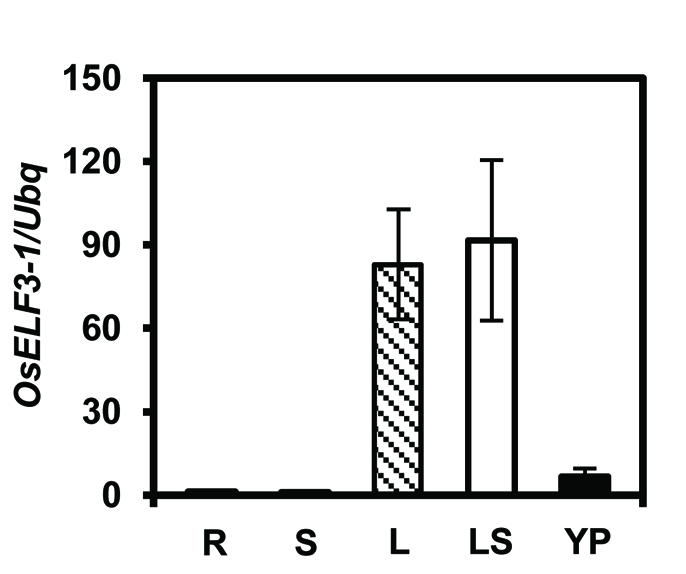

Supplement: Figure S3 — Tissue-specific expression of OsELF3-1. QRT-PCR analysis of OsELF3-1 expression in different tissues. Rice were grown in Sichuan natural LD field and harvested at 9∶00 am at the booting stage. Values are means ± SD. (TIF) [file pone.0043705.s003.tif]

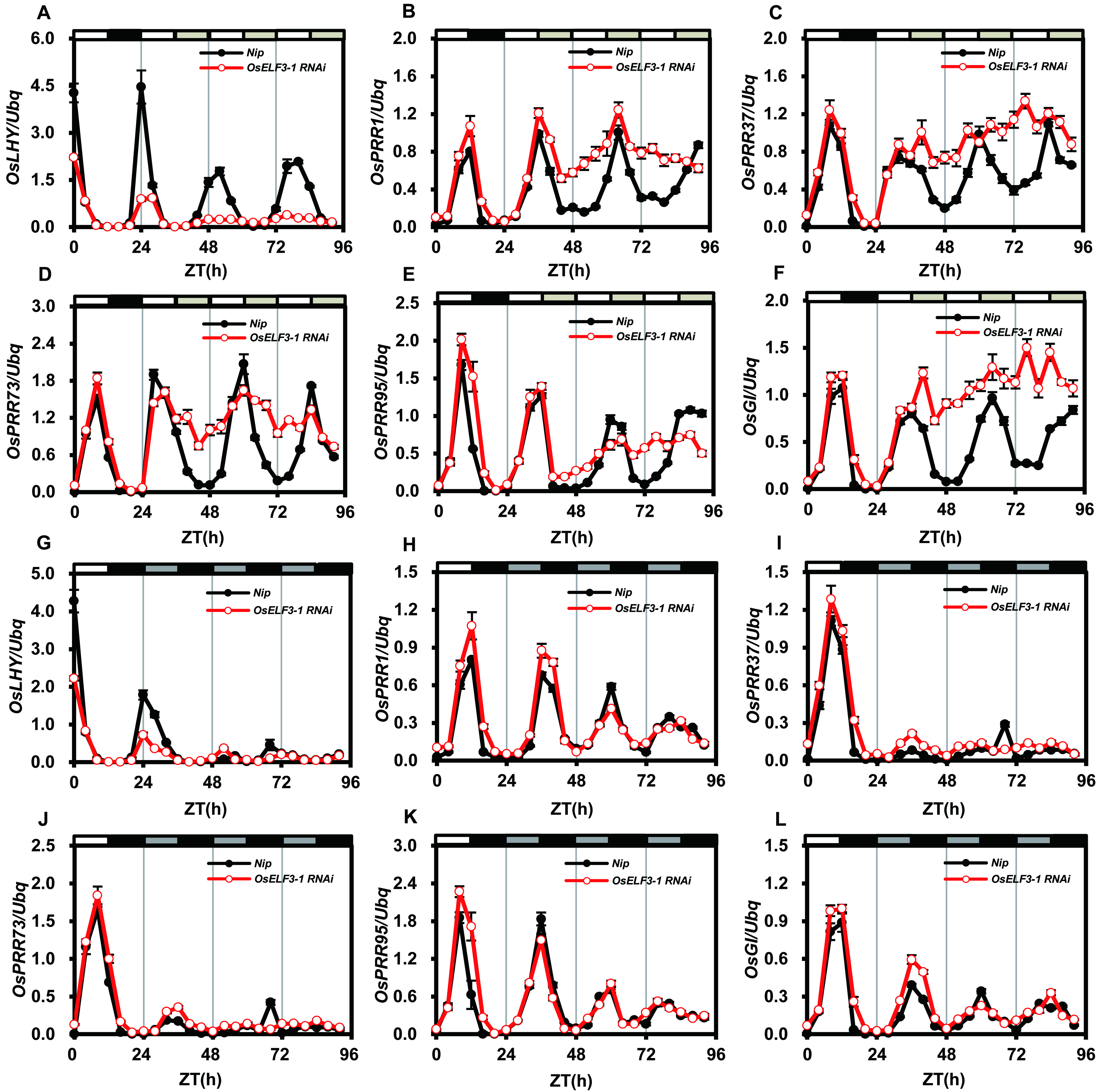

Supplement: Figure S4 — Circadian expression of clock-associated genes in Nip and OsELF3-1 RNAi. Circadian expression of OsLHY, OsPRR1, OsPRR37, OsPRR73, OsPRR95 and OsGI in Nip (filled circles) and OsELF3-1 RNAi (open circles) under LL and DD. The plants were first grown to cycles of 12-hour light/12-hour darkness for 30 days at 26°C and then transferred to the continuous light (LL) or continuous darkness (DD) at dawn. In A-F panels, white bars, light; black, darkness; gray, subjective light. In G-L panels, white bars, light; black, darkness; gray, subjective darkness. ZT means Zeitgeber time. Values are means ± SD. (TIF) [file pone.0043705.s004.tif]
