# Supplementary material for: OsELF3-1, an Ortholog of Arabidopsis EARLY FLOWERING 3, Regulates Rice Circadian Rhythm and Photoperiodic Flowering
Source: PLoS One. 2012 Aug 17;7(8):e43705. doi: 10.1371/journal.pone.0043705 (PMC3422346; doi:10.1371/journal.pone.0043705)
Supplement: Table S1 — Days to heading in oself3-2 plants in 2010 and 2011. Days to heading was calculated when the first panicle appeared. WT means wild type. (DOCX) [file pone.0043705.s005.docx]

Days to heading was calculated when the first panicle appeared. WT means wild type.

| **Genotypes** |  | **Days to heading in 2010** | | |  | **Days to heading in 2011** | |
| --- | --- | --- | --- | --- | --- | --- | --- |
|  |  | **Wenjiang Hainan** | | |  | **Wenjiang Hainan** | |
| WT (*ZH11*) |  | 86 ± 1.0 71 ± 4.9 | | |  | 88 ± 1.0 57 ± 1.9 | |
| *oself3-2* (*ZH11*) |  | 85 ± 1.3 69 ± 3.0 | | |  | 87 ± 1.3 63 ± 2.7 | |
| WT (*DJ*) |  |  |  |  |  | 87 ± 0.9 |  |
| *oself3-2* (*DJ*) |  |  |  |  |  | 89 ± 0.5 |  |
