# Supplementary material for: OsELF3-1, an Ortholog of Arabidopsis EARLY FLOWERING 3, Regulates Rice Circadian Rhythm and Photoperiodic Flowering
Source: PLoS One. 2012 Aug 17;7(8):e43705. doi: 10.1371/journal.pone.0043705 (PMC3422346; doi:10.1371/journal.pone.0043705)
Supplement: Table S2 — Primers used in this study. (DOCX) [file pone.0043705.s006.docx]

| **Primer** | **Forward 5’- 3’** | **Reverse 5’- 3’** |
| --- | --- | --- |
| ***HPT*** | TACACAGCCATCGGTCCAGA | TAGGAGGGCGTGGATATGTC |
| **LP** | AAGTGCGAATGGCAAAAGTC |  |
| **RP** |  | TTTCCTCTATGCCTCCTTGC |
| **sense** | GCTCTAGAACTAAGGGCATGGATATTTATGCTTC (*Xba*I) | CCCGGATCCTCATTTCTTCTCATTGGAGGGTTCTG (*Bam*HI) |
| **antisense** | GCGTCGACACTAAGGGCATGGATATTTATGCTTC (*Sal*I) | CCCAAGCTTTCATTTCTTCTCATTGGAGGGTTCTG (*Hind*III) |
| ***qUbq*** | AACCAGCTGAGGCCCAAGA | ACGATTGATTTAACCAGTCCATGA |
| ***qOsELF3-1*** | TGTCGCCCCTTCGTCAA | GGTCTTTTCCCCAGCTCATT |
| ***qOsELF3-2*** | GCTGGATGGCATTTGACTG | ATTTGCGACTTTGAGGGACTA |
| ***qOsPRR37*** | GGGAAGGACTTGGAAATAG | AGCAGCTCGAACACTTGACT |
| ***qOsPRR73*** | AGGAGCGGAAAGAAACATAA | AACCTTGGAGGAGCAATCAG |
| ***qOsPRR95*** | CGCTCAGTGGCAGTGTCTGT | GGTATCGCACCTTCTTCTCA |
| ***qOsGI*** | TGGAGAAAGGTTGTGGATGC | GATAGACGGCACTTCAGCAGAT |
| ***qOsLHY*** | CAGATAAGGCCGACACCAAAC | GGTGTGTTGGAACCACATG |
| ***qGhd7*** | ATATTGTGGGAGCACGTT | ATCTGAACCATTGTCCAAGC |
| ***qEhd1*** | GCGCTTTTGATTTCCTGC | TTCGGAATATGTGCTGCC |
| ***qHd3α*** | GCTCACTATCATCATCCAGCATG | CCTTGCTCAGCTATTTAATTGCATAA |
| ***qRFT*** | TACTTCAACTGCCAGCGCGAGG | AGCTATAGCTGCTGCATGCATGGA |

**Note: The underlined nucleotides indicate the restriction sites for cloning.**
